# A review of the genetic spectrum of hereditary spastic paraplegias, inherited neuropathies and spinal muscular atrophies in Africans

**DOI:** 10.1186/s13023-022-02280-2

**Published:** 2022-03-24

**Authors:** Amokelani C. Mahungu, Nomakhosazana Monnakgotla, Melissa Nel, Jeannine M. Heckmann

**Affiliations:** 1 Neurology Research Group, University of Cape Town Neuroscience Institute, Cape Town, South Africa; 2 E8-74 Neurology, Department of Medicine, Groote Schuur Hospital and the University of Cape Town Neuroscience Institute, University of Cape Town, Cape Town, South Africa

**Keywords:** Hereditary spastic paraplegia, Genetic neuropathies, Charcot Marie Tooth disease, CMT, Spinal Muscular Atrophy, Africa, Inherited neuromuscular disorders

## Abstract

**Background:**

Genetic investigations of inherited neuromuscular disorders in Africans, have been neglected. We aimed to summarise the published data and comment on the genetic evidence related to inherited neuropathies (Charcot-Marie-Tooth disease (CMT)), hereditary spastic paraplegias (HSP) and spinal muscular atrophy (SMA) in Africans.

**Methods:**

PubMed was searched for relevant articles and manual checking of references and review publications were performed for African-ancestry participants with relevant phenotypes and identified genetic variants. For each case report we extracted phenotype information, inheritance pattern, variant segregation and variant frequency in population controls (including up to date frequencies from the gnomAD database).

**Results:**

For HSP, 23 reports were found spanning the years 2000–2019 of which 19 related to North Africans, with high consanguinity, and six included sub-Saharan Africans. For CMT, 19 reports spanning years 2002–2021, of which 16 related to North Africans and 3 to sub-Saharan Africans. Most genetic variants had not been previously reported. There were 12 reports spanning years 1999–2020 related to *SMN1*-SMA caused by homozygous exon 7 ± 8 deletion. Interestingly, the population frequency of heterozygous *SMN1*-exon 7 deletion mutations appeared 2 × lower in Africans compared to Europeans, in addition to differences in the architecture of the *SMN2* locus which may impact *SMN1*-SMA prognosis.

**Conclusions:**

Overall, genetic data on inherited neuromuscular diseases in sub-Saharan Africa, are sparse. If African patients with rare neuromuscular diseases are to benefit from the expansion in genomics capabilities and therapeutic advancements, then it is critical to document the mutational spectrum of inherited neuromuscular disease in Africa.

**Highlights:**

- Review of genetic variants reported in hereditary spastic paraplegia in Africans
- Review of genetic variants reported in genetic neuropathies in Africans
- Review of genetic underpinnings of spinal muscular atrophies in Africans
- Assessment of pathogenic evidence for candidate variants

**Supplementary Information:**

The online version contains supplementary material available at 10.1186/s13023-022-02280-2.

## Introduction

Inherited neurological diseases in African populations have been largely neglected. Africans will be left behind in the global quest for targeted genetic therapies without an African perspective on disease-associated mutations. While modern genomic approaches have led to new gene discoveries in complex inherited neuromuscular disorders [1], the genetic landscape of neuromuscular disorders in Africans are barely known.

Inherited neuromuscular disorders, such as hereditary spastic paraplegia (HSP) and Charcot-Marie-Tooth (CMT) disease are not rare in North America, Europe, and Asia with a global prevalence ranging between 4.3/100,000 for HSP and 82.3/100,000 for CMT [1, 2]. There are no epidemiological data for Africa. Akinyemi et al. reported that of the 58 African states, scattered reports related to the genetics of neurological disorders emanated from only 17 countries and these were heavily concentrated in four North African countries [3]. Presently in South Africa, and with relevance to this review, the National Health Laboratory Service offers one genetic screening test for CMT (the common *PMP22* gene duplication/deletion) and none for HSP. Although the screening test to detect the most common cause of Spinal Muscular Atrophy (SMA) (homozygous deletion/disruption of *SMN1*) has been available in South Africa for more than 2 decades, only isolated cases are able to access gene therapies for SMA which are available in resource-rich countries. Therefore, there is an urgent need to address the disparate healthcare in inherited neuromuscular diseases which exist between the developed world and Africa. However, there are presently a few initiatives such as the International Centre for Genomic Medicine in Neuromuscular Diseases (ucl.ac.uk/genomic-medicine-neuromuscular- diseases/) to prioritise the advancement of genetic research in neuromuscular diseases, and the broader H3Africa Initiative to expand population reference data in sub-Saharan Africans [4], which will facilitate the analysis of pathogenic genetic variants in Africans with rare inherited diseases. This review will synthesize genetic reports from HSP, CMT and SMA in Africans, to give an overview of the genetic variants and their associated phenotypes, which have been reported and can be used as a reference resource for African researchers and clinicians. A separate review of inherited myopathies and muscle dystrophies in Africans, is in progress.

## Methodology

**Fig. 1:**
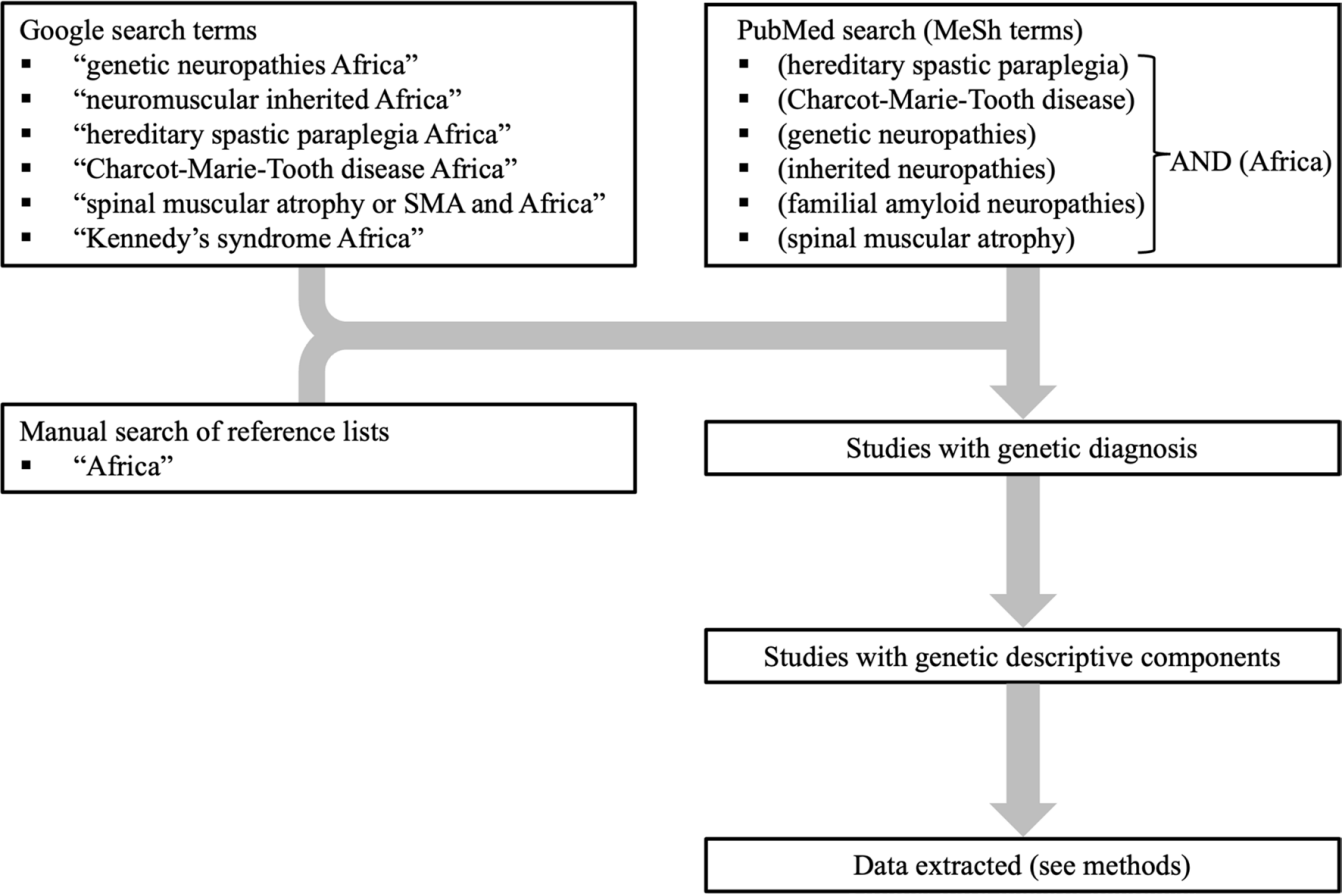
Flow chart describing the methodology used for the literature curation

PubMed was searched for journal articles related to the molecular genetic causes of HSP, CMT, and SMA in Africa. The following MeSH terms were used (hereditary spastic paraplegia) or (Charcot-Marie-Tooth disease) or (genetic neuropathies) or (inherited neuropathies) or (familial amyloid neuropathies) AND (Africa), and (spinal muscular atrophy) AND (Africa) for searching PubMed. We performed a google search using search terms: “genetic neuropathies Africa”, “neuromuscular inherited Africa”, “hereditary spastic paraplegia Africa”, “Charcot-Marie-Tooth disease Africa”, “spinal muscular atrophy or SMA and Africa”, “Kennedy’s syndrome Africa”. We also manually searched the reference lists of reports and review publications to look for additional references and searched for “Africa” within articles. We confined this review to studies with genetic descriptive components. Studies involving linkage analysis of a large genomic region or single genes where a genetic diagnosis was not reached were excluded as the focus of this paper was on the identified genetic causes of inherited neuromuscular disorders in Africans (European or Indian ancestries were excluded) (Fig. 1). Reports related to infectious disease-associated neuropathies were excluded. Only English articles were reviewed which resulted in the exclusion of two reports from 2002 and 2008 which were published in French.

The data collected from the reports included: the genetic results of probands with African-genetic ancestry, phenotypic features including age at onset, inheritance pattern and consanguinity, and electrophysiological features. We also noted genetic variants found in Africans but which had been previously reported in non-African families, whether there were attempts to determine segregation of the putative disease-causing variant within the family, and whether population controls were assessed for the variant. Segregation of genetic variation was scored positive if the putative disease-causing variant was (a) excluded in at least one unaffected individual of the same age or older than the affected individual for autosomal dominant inheritance, or (b) confirmed in the heterozygous state in at least one unaffected parent for autosomal recessive inheritance. Variants in which functional studies had been performed were noted. In addition, as many of these publications were published prior to the establishment of large scale public genetic databases, we also interrogated the gnomAD database (last accessed 6 Sept. 2021) to determine the frequency of putative disease-causing variants [5]. For variant nomenclature we followed the Human Genetic Variation Sequence (HGVS)(version 20.05) guidelines [6].

## Results

Most reports used the following genetic methodologies: Targeted PCR sequencing and/or Sanger sequencing; multiplex ligation-dependent probe amplification; and HSP or CMT gene panels. Some studies used appropriate microsatellite markers to construct segregating haplotypes to establish linkage in families followed by targeted Sanger sequencing of coding exons. More recent reports (from 2013) used whole exome sequencing (WES) to screen protein coding variants or  performed comprehensive whole genome sequence (WGS) analysis.

### Hereditary spastic paraplegia

Hereditary spastic paraplegias (HSP) are clinically characterized by a progressive gait disturbance due to increasing spasticity of the legs. Clinicians have recognized two forms of HSP; patients who only have features of HSP (or pure HSP), or those with additional neurological system dysfunction such as ataxia, cognitive/intellectual disability, extrapyramidal signs, and features of sensory ± motor neuropathy. The latter are called complex HSP.

Although the clinical manifestations of HSP usually manifest over years rather than months, it remains important to exclude other non-degenerative conditions by performing imaging studies of the brain and spinal cord. Magnetic resonance imaging (MRI) of the brain may be normal or show atrophy, and/or may show thinning of the corpus callosum and/or increased white matter signal intensities (Table 1). In Africa, infectious causes such as HTLV1-associated tropical spastic paraparesis is a concern in adults, which can be excluded with cerebrospinal fluid examination and/or serology [7]. Lathyrism caused by excessive consumption of the chickpeas of the lathyrism family, is endemic in Ethiopia, and can result in a slowly progressive paraparesis [7].

**Table 1:** Genetic causal variants of HSP identified in African populations

Ref	Country	HSP type	Inh	AAO, years	Additional phenotypic features	Gene	HGVS	Gene Variant assessment		
								Proband count	Segregation	Pop. freq
Northern Africa										
[26]	Tunisia^+^	SPG5	AR	9–10	WM-HI	*CYP7B1*	R112* ^a^	1	Yes	No
[14]	Morocco^−^, Algeria^+^	SPG7	AR	~ 30 < 10	-	*PGN*	F284fs/V581del^b^ Q82del	1 1	Yes No	Yes Yes
[9, 15–17, 25]	Algeria^±^, Morocco^+^, Tunisia^+^, Egypt^+^ Sudan^+^	SPG11	AR	2–23	± dysarthria/dysphagia; ± Cog; ± scoliosis; ± pes cavus; UL tremor; ± weakness/atrophy UL/LL; ± ataxia; ± epilepsy; ± TCC/WM-HI; ± motor axonopathy	*KIAA1840*	R2034* ^c^ M245fs ^a,c^ V2344fs S412L L517fs Q498*^a^ K1190* G2117* A2237fs c.5866 + 1G > A^c^	10 5 1 1 1 2 1 1 2 1	Yes Yes Yes Yes Yes Yes Yes Yes Yes Yes	Yes Yes Yes Yes Yes Yes Yes Yes Yes Yes
[18, 25]	Tunisia^+^, Morocco^+^, Algeria^+^	SPG15	AR	1– 20	± Cog, ± PBD, pes cavus, ± scoliosis, ± LL atrophy, ± TCC/WM-HI, ± axonopathy	*ZFYVE26*	S2004T Q493* F683fs R1438* ^a,c^ c.5485-1G > A	1 4 2 1 1	Yes Yes Yes Yes Yes	No Yes Yes Yes Yes
[22]	Tunisia^+^ Algeria^+^	SPG26	AR	3–19	Cog., ataxia, PNP; WM-HI	*B4GALNT1*	R300C^c^ L89fs	1 1	Yes No	Yes Yes
[23]	Morocco^+^	SPG28	AR	< 1	Cog., WM-HI/BG calcification	*DDHD1*	R589Q	1	Yes	Yes
[20]	Morocco^+^	SPG35	AR	4	Cog	*FA2H*	G46D	1	No	No
[20]	Morocco^+^	SPG48	AR	< 1	Cog., ataxia	*AP5ZI*	R206W	1	Yes	No
[21]	Tunisia^+^	SPG46	AR	2–10	Cog., ataxia, cataracts	*GBA2*	R630W	1	Yes	Yes
[13]	Morocco^+^	SPG51	AR	< 1	Cog., PBD	*AP4E1*	R1105* ^c,d^	1	Yes	Yes
[9]	Sudan	SPG57	AR	< 1.2	± Microcephaly	*TFG*	R22W ^c,d^	1	Yes	Yes
[9]	Sudan	ARSACS	AR	10–11	± weakness UL/LL; ± ataxia; ± Cog.; SM axonopathy	*SACS*	W2580*	1	Yes	Yes
[25]	Morocco^+^	UK	AR	1–5	Ulcero-mutilating neuropathy; SM axonopathy	*CCT5*	H147R	1	Yes	Yes
[12]	Tunisia^+^	UK	AR	2	Optic atrophy	*RNF170*	delEx4_7 ^d^	1	Yes	Yes
[10]	Morocco^+^	SPG76	AR	20–39	± Dysarthria; ± ataxia; ± pes cavus; scoliosis; PNP	*CAPN1*	R295P G527*	1 1	Yes Yes	Yes Yes
[11]	Egypt	SPOAN	AR	< 1	Optic atrophy; neuropathy	*KLC2*	216bpdel 5’UTR ^a,d^	1	UK	Yes
[9]	Sudan	UK	AR	< 1.5	± PBD^c^	*ALS2*	C123Y	1	Yes	Yes
[9]	Sudan	SPG3A	AD	1.5–7	± proximal weakness LL	*ATL1*	F151S	1	Yes	Yes
[25, 27, 28]	Morocco^−^ Tunisia^−^	SPG4	AD AD S	10–20 12–38 1	± Cog	*SPAST*	R499C ^a^ S404F G442K	> 2 1 1	Yes Yes Yes	Yes Yes Yes
Sub-Saharan Africa										
[17]	Kenya^+^	SPG7	AR	~ 30	Ataxia	*PGN*	L78 ^c^	1	No	No
[17, 32]	Kenya ^+^ Somalia^−^	SPG11	AR	10–20 ~ 2	Oromandibular dystonia ± Cog; ± ataxia	*KIAA1840*	S1923fs ^c^ A2237fs	3 1	No No	No No
[29]	Mali^+^	SPG35	AR	~ 2	dysphagia	*FA2H*	c.786 + 1G > A ^a^	1	Yes	No
[31]	Mali^+^	SPG43	AR	7–12	SM neuropathy	*C19orf12*	A63P ^a,c^	1	No	Yes^e^
[33]	South Africa^−^	SPG3A	AD	50–60	Cog.; TCC	*ATL1*	R416C ^c^	1	Yes	Yes
[30]	Mali^+^	SPG10	AD	10–20	SM neuropathy; axonopathy	*KIF5A*	K362N	1	Yes	Yes

*AAO* age of onset, *Inh* inheritance pattern, *AD* autosomal dominant, *AR* autosomal recessive, *S* sporadic, *cog* cognitive abnormalities, *SM* neuropathy refers to sensori-motor polyneuropathy, *PNP* peripheral neuropathy unspecified, ‘axonopathy’ refers to electrophysiological studies showing axonal loss (either motor and/or sensory); *UL* upper limb, *LL* lower limb, *PBD* pseudobulbar dysarthria reflecting spastic dysarthria and emotional incontinence;—no additional signs other than CMT; ^+^, consanguinity; ^−^, no consanguinity; WM-HI refers to brain MRI findings of white matter hyperintense signal changes; TCC, thinning of corpus callosum; UK, unknown; SPOAN, spastic paraplegia, optic atrophy and neuropathy; HGVS, Human Genome Variation Society protein (p.) level and splice-site coding (c.) level recommendations (version 20.05)

Gene Variant score: Proband count, number of probands investigated by study; Segregation—yes when the pathogenic variant segregation was shown within the family (see methods). Pop. freq., Population frequency- yes when controls in the same population were assessed

^a^Variant has been reported in non-African probands/families

^b^Compound heterozygous variant

^c^Present in gnomAD v2/v3 (see Additional file: for frequencies)

^d^Functional studies for variant was performed

More than 88 genes have thus far been reported to cause HSP, which are designated as SPastic Gait/Gene or SPG genes [2, 8]. Inheritance patterns in HSP are predominant autosomal dominant (AD), except in areas with high consanguinity, such as in North Africa, where autosomal recessive inheritance (AR) patterns are prevalent [2, 8, 9] (See Table 1). X-linked and mitochondrial maternal inheritance patterns of HSP are rare [8]. World-wide SPG4 is reported to account for up to 79% of HSP cases with AD inheritance, albeit mainly in those with Caucasian ancestry [8]. Other frequent causes of AD HSP include the monoallelic pathogenic variants in *KIF1A,* as well as SPG3A and SPG31 [8]. Genes accounting for HSP cases with AR inheritance patterns include SPG11 and SPG7, followed by SPG15 and SPG5 in overall frequencies [8]. Interestingly, three genes (*KIF1C*, *SPG7*, *KIF1A)* have been reported to associate with mixed inheritance patterns related to allele-dose-dependent clinical phenotypes i.e. milder phenotypes with heterozygous variants, and more severe phenotypes with homozygous states [8].

#### HSP in North Africa

**Fig. 2:**
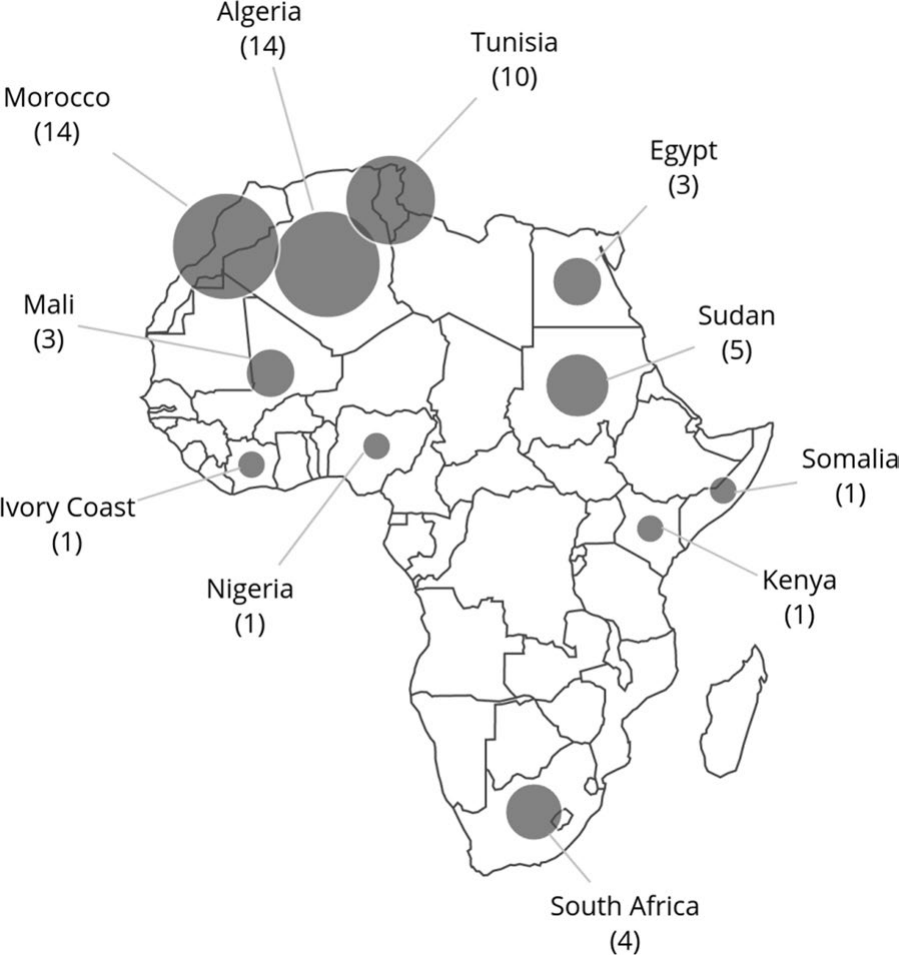
Bubble map depicting the number of genetic reports in HSP- and CMT-related disorders in African countries

Most of the genetic reports on HSP in Africa are from North Africa and are based on targeted linkage analysis in families to identify a candidate gene locus that segregated with the phenotype, followed by direct gene sequencing (Fig. 2). The commonest gene harbouring a pathogenic variant identified in HSP cases, was *SPG11* (*KIAA1840*) associated with thin corpus callosum on MRI [9] (Table 1; Additional file 1: Table A). Only four of the reports used WES for a more comprehensive gene screen in North African cases with HSP conditions, and one used WGS data [10–13]. The commonly encountered AR-HSP causal genes (*SPG11, SPG15*) in North African populations were also amongst the top seven genes in a large European cohort [9, 10, 14–19]. Other AR-HSP genes amongst North African families included *SPG5, SPG7, SPG35, SPG46, SPG48, SPG51,* and *SPG57,* as well as mutations in the *ALS2* and *SACS* genes [9, 13, 14, 20, 21] However, private mutations in novel genes (*RNF170*, *CAPN1, KLC2, B4GALNT1, DDHD1, CCT5)* were also reported to be disease-causing in isolated cases or families [10–12, 21–25].

The most frequent gene variants accounting for autosomal dominant inheritance patterns, were found in *SPG4* (*SPAST*) [26–28].

#### HSP in sub-Saharan Africa

Six reports were found from sub-Saharan Africa of which two screened a targeted panel of 58 HSP genes [29, 30] and two used WES [17, 31] (Table 1; Additional file 1: Table A; Fig. 2). The cases from consanguineous families from Kenya and Mali with homozygous pathogenic alleles were most frequent with *SPG11* variants, followed by *SPG7*, *SPG35* and *SPG43* [32].

There were two reports on autosomal dominant HSP; one black South African family with a novel *SPG3A* variant [33] and a family from Mali with *SPG10* [30]. Therefore, the common SPG genes present in Europeans [19], viz. *SPG3*, *SPG4* and *SPG10*, have been found in isolated African cases.

### Genetic neuropathies

The largest group of genetic neuropathies are referred to as the Hereditary Sensory Motor Neuropathies or Charcot Marie Tooth (CMT) disease. CMT affects predominantly the motor and sensory nerves, although the CMT-spectrum includes rare forms with autonomic and motor only involvement [34]. The clinical features of CMT disease are progressive and symmetrical weakness and wasting of distal muscles of the foot and ankle which may result in clumsy feet, foot deformities such as pes cavus, and loss of deep tendon jerks. Later, there may be involvement of the distal arms with wasting and weakness although clawing of the hands is less common. Some genetic neuropathies may have early and predominant upper limb involvement. Sensory involvement ranges from mild distal numbness to severe loss of sensation with ulcers, and/or sensory ataxia. The insidious clinical progression of CMT distinguishes it from subacute acquired inflammatory neuropathies in most cases, although rare forms of CMT can give a patchy electrophysiological picture with conduction blocks that may resemble treatment-resistant chronic inflammatory demyelinating polyradiculoneuropathy [34]. In Southern African populations, where HIV-infection is prevalent, small fibre painful neuropathies may be considered in cases with more advanced HIV-infection, and/or with concomitant tuberculosis and isoniazid therapies, but weakness is extremely rare [35]. This contrasts with CMT where the absence of motor involvement is unlikely [34].

In the pre-molecular era, CMT was categorized by the electrophysiological involvement of the sensory and motor nerves, whereas the CMT neuropathies are further categorized according to their electrophysiological findings into three types; the demyelinating forms (nerve conduction velocities (NCVs) < 38 m/s in the upper limbs), axonal forms (NCV > 45 m/s), or the intermediate types of CMT (NCV in the upper limbs between 25 and 45 m/s) [1]. All neuropathies categorized as HSMN or CMT, would show evidence of motor and sensory nerve abnormalities on electrophysiological testing, whereas hereditary motor neuropathy (HMN) by definition would have normal sensory nerve action potential responses. However, there appears to be genetic overlap between CMT2 and HMN subtypes [36].

In North America and European populations, most CMT neuropathies show AD inheritance compared to AR inheritance which comprises < 10% of cases. In contrast, in North Africa, where consanguinity is high [37], most of the cases published showed AR inheritance (Table 2). Similar to what is observed in HSP, CMT shows substantial genetic heterogeneity with > 100 genes identified which can cause genetic neuropathies [1]. The most common autosomal dominantly inherited CMT in North America and Europe, the demyelinating CMT1A caused by a duplication in the *PMP22* gene, accounts for ~ 40% of genetic neuropathies [38], yet remains unreported in those with African genetic ancestry.

**Table 2:** Genetic causal variants of Charcot-Marie-Tooth (CMT)-related disorders reported in African populations

Ref	Country	Disease	Inh	AAO, years	Phenotypic features in addition to CMT	Gene	Gene variant	Gene Variant Assessment		
								Proband count	Segregation	Pop Freq
**North Africa**										
[24, 39–41]	Algeria^±^ Morocco^+^	CMT2B1	AR	2 – 27	± proximal LL weak; ± scoliosis; axonopathy	*LMNA*	R298C^a^	28	Yes	Yes
[24, 42, 48, 49, 51]	Morocco^+^ Tunisia^+^ Morocco^−^	CMT4A	AR	< 2 1–6 3	± kyphosis; claw hands; ± proximal LL weak; demyelinating ± proximal LL weak; claw hands; axonopathy ± proximal LL weak; ± diaphragm; axonopathy	*GDAP1*	W31* P78L (S194*^b^) R161H S194* ^a,c^(R310Q^b^)	2 3 1 8	No Yes No Yes	No Yes Yes Yes
[37]	Algeria^+^	CMT4B1	AR	1 – 12	Chest deformity; claw hands; ± vocal cord paralysis; demyelinating	*MTMR2*	p.R111fs	1	Yes	Yes
[47]	Tunisia^+^/Morocco^+^	CMT4B2	AR	2 – 15	± Glaucoma; demyelinating	*MTMR13*	R1196*^a^ Q956*	1 1	Yes Yes	Yes Yes

Ref	Country	Disease	Inh	AAO	Phenotypic features in addition to CMT	Gene	Gene variant	Proband	Segr	P Freq
[50]	Algeria	CMT4C	AR	4–10	Scoliosis; ± cranial neuropathy (hypoacusia/facial); demyelinating	*SH3TC2*	E731fs (Het) c.1178-1G > A R904*^a^ R954* ^a^	1 1 1 1	No No Yes No	No No No No
[37]	Algeria^+^	CMT4F	AR	10–12	Kyphoscoliosis; ± sensory ataxia; demyelinating	*PRX*	p.Arg364Ter	1	Yes	Yes
[43–46]	Tunisia^+/^ Algeria^+^	CMT4H	AR	< 2	Scoliosis; “Ataxia”; demyelinating	*FGD4*	A172fs M298T R442H	1 1 1	No Yes Yes	Yes Yes Yes
[52, 53]	Algeria^−^	dHMN	AD	11–35	UL motor axonopathy	*GARS*	G526R	4	Yes	No
**Sub-Saharan Africa**										
[54]	Nigeria^−^	CMT1B	AD	> 50	Demyelinating	*MPZ*	S78W	1	No	Yes
[56]	Mali^+^	CMT2D	AR	12	UL motor/sensory; ± seizures; S/M axonopathy	*GARS*	S265Y (Het)	1	No	No
[55]	Ivory Coast^+^	CMTint	AR	< 10	Proximal weak; MRI-WM; raised CK; conduc. blocks	*PLEKHG5*	C35fs	1	Yes	Yes

AAO, Age of Onset (years); Inh, inheritance pattern; AD, Autosomal dominant; AR, Autosomal recessive; S, sporadic; cog, cognitive abnormalities; SM neuropathy refers to sensori-motor polyneuropathy; PNP, peripheral neuropathy unspecified; ‘axonopathy’ refers to electrophysiological studies showing axonal loss (either motor (M) or sensory (S)) or ‘demyelinating’ slowing of conduction velocities; conduc. blocks refers to conduction blocks at unusual sites on electrophysiological testing; UL, upper limb; LL, lower limb; CMTint. refers to intermediate CMT (or distal spinal muscular atrophy type 4/DSMA4); dHMN or distal hereditary motor neuropathy (also classified as DSMA5); MRI-WM white matter signal changes on brain MRI; ^+^, consanguinity; ^−^, no consanguinity; HGVS, Human Genome Variation Society protein (p.) level and splice-site coding (c.) level recommendations (version 20.05)

Gene Variant score: Proband count, number of probands per variant; Segregation – yes when the pathogenic variant segregation was shown within the family (see methods); Pop. freq., Population frequency- yes when there was an attempt at assessing controls in the same population

^a^Variant has been reported in non-African probands/families

^b^Compound heterozygous; *GDAP1* S194* was reported as a compound heterozygous variant in two families with P78L and R310Q, respectively

^c^Present in gnomAD v2/v3 (see Additional file: table for frequencies)

#### CMT in North Africa

Due to high levels of consanguinity in Algeria, Morocco, and Tunisia, AR-CMTB1 (*LMNA)* was by far the commonest, followed by CMT4A (*GDAP1),* CMT4C (*SH3TC2*), and CMT4B2 (*MTMR13*) [24, 39–50](Table 2). These are present in < 1% of AR-CMT cases in non-Africans [38]. Two Algerian families had compound heterozygous pathogenic variants with the common *GDAP1* S194* variant [51], which has a population frequency of 2.3 × 10^–5^ (Additional file 1: Table B). Isolated cases were reported with CMT4B1 and CMT4F [37].

Four Algerian families with distal HMN (dHMN5A) and AD inheritance patterns were reported with the rare [38] *GARS* pathogenic variants characterised by predominant upper limb weakness and hand wasting [52, 53].

#### CMT in Sub-Saharan Africa

Three reports were found (Fig. 2). One CMT1B (*MPZ*) Nigerian AD pedigree with late-onset demyelinating neuropathy [54]; and an intermediate CMT phenotype with conduction blocks and a novel *PLEKHG5* variant which segregated in the family [55]. A consanguineous pedigree from Mali was reported with a heterozygous *GARS* variant, but without evidence of segregation or population screening [56]. Caution must be used in interpreting variants with “incomplete penetrance” to explain incomplete segregation of variants particularly in Africans where the population data are sparse and genetic variation is increased [57].

#### Familial amyloid neuropathies

There are three types of familial amyloid neuropathies (FAP) which are categorised according to the abnormal precursor protein which will result in downstream deposition of amyloid fibrils viz. transthyretin (TTR), apolipoprotein A-1 and gelsolin [58]. Although some *TTR* mutations can cause FAP, which characteristically manifests with sensory and autonomic nerve dysfunction alone, a rare manifestation is oculoleptomeningeal amyloidosis (OLMA) which may present with additional features such as subarachnoid haemorrhage, epilepsy, hearing and visual loss, and headaches [59]. OLMA was described in a Nigerian adult heterozygous for *TTR* L21P*,* a variant which was previously reported in several European-ancestry cases [59]. Another common variant, at least among African-Americans (and found amongst West Africans), is the *TTR* V122I variant which was detected in the heterozygous state in 4% of African-Americans [60] and is associated with hypertrophic restrictive cardiomyopathy in older individuals, but without neuropathy. A man from Benin was reported with cognitive changes, a sensori-motor neuropathy with autonomic involvement and sensory ataxia, as well as hypertrophic cardiomyopathy, and a *TTR* I107V variant, which has been found in several Europeans with inherited amyloidosis [61].

### Spinal muscular atrophies

Classical Spinal Muscular Atrophy (SMA) due to the homozygous loss of exon 7 (± exon 8) of *SMN1* results in a critical loss of protein production and progressive degeneration of the lower motor neurons of the spinal cord [62]. We will refer to this as *SMN1*-SMA. Clinically, *SMN1*-SMA is characterized by proximal muscle atrophy and weakness, and eventually distal paresis as well. The clinical subtypes of *SMN1*-SMA (types I–IV) were categorized based on the disease severity and age at onset, which also informed the prognosis and survival; Type I is most severe and manifests in early infancy, SMA II manifests in late infancy to early childhood (< 18 months), SMA III in childhood (> 18 months)[62] and SMA IV has adult-onset [63].

There is increasing recognition of *SMN1*-negative SMA, although this groups accounts for < 5% of SMA and is often associated with overlapping central nervous system/brainstem signs, and even cardiomyopathy [63]. However, in reports from Africa there are between 25 and 65% of the clinical cohorts categorised as either congenital hypotonia or SMA phenotypes, which can be categorized as *SMN1-*negative SMA (absence of homozygous exon 7 deletion). In addition, there are several types of distal SMA (DSMA) which overlap with classifications of distal HMN/dHMN [63] (see Table 2).

The *SMN2* gene is a highly homologous centromeric copy of *SMN1* in which a C > T variant in exon 7 splicing enhancer distinguishes *SMN2* from *SMN1* [64]. Although genetic variation in *SMN2* does not cause disease, *SMN2* copy numbers may modify disease severity and age at onset [65].

#### SMN1-SMA in North Africa

*SMN1-*SMA in North African populations have been reported in families with and without high consanguinity rates [66–75] (Additional file 2: Table C). Similar to European cohorts, 57/60 (95%) Tunisian cases with presumed *SMN1*-SMA showed homozygous deletion of *SMN1* exon 7 [70], although the other samples showed lower proportions of *SMN1*-SMA particularly in older individuals [69].

#### SMN1-SMA in sub-Saharan Africa

Five reports on SMA in sub-Saharan Africans were found, mostly involving South Africans and one each from Congo and Mali (Additional file 2: Table C) [73–75]. Several cases from two regions in South Africa reported *SMN1*-SMA with homozygous loss of exon 7 (± exons 8) ranging between 35 and 100% of their clinical samples, indicating a substantial number of cases with an alternative molecular diagnosis [76–78]. An *SMN1* gene dosage assay in 300 random black SA samples showed the heterozygote exon 7 deletion in 6 individuals (1/50 population controls; 2%) which was similar to the frequency of *SMN1* copy numbers in Kenyans and Nigerians [74], but roughly half of the heterozygote frequency found in European ancestry controls (3–4%)[77]. In comparison, the heterozygote frequency amongst 628 Malians was found to be 0.5% [74].

Humans have variable copies of an *SMN2* gene, between 0 and 8 copies, and transcripts of this gene can modify the expression of *SMN1*-SMA [63]. Interestingly, the architecture of the *SMN* region differs substantially between Europeans and Africans, although African-Americans roughly followed the same trends in terms of *SMN2* copy numbers as Europeans and Asians [79]. Amongst 75 black South African *SMN1-*SMA patients, 11% had > 2 *SMN2* copies compared with 37% (of 30) *SMN1-*SMA patients with European ancestry [78]. Taken together, these results underscore the fact that the genetic architecture and disease pathogenic mechanisms in African ancestry individuals may vary from Europeans, and requires further study.

### Complex inherited conditions with neuromuscular features

Although there are numerous complex multi-system conditions in which the presence of neuropathy may be present but not prominent [34], we mention two reports in Africans in which the recognition and initiation of appropriate treatment underscores their importance. Two families/probands with Allgrove or Triple A syndrome was described from North Africa/Algeria with the homozygous pathogenic variant in the *AAAS* gene (IVS14 + 1G > A); 1 family was consanguineous [80] and in the other both parents were heterozygous for the variant [81]. The main features were ACTH-resistant adrenal deficiency, achalasia and dry eyes, as well as features of distal motor neuropathy with/without spasticity, with the clinical onset during childhood. The importance is to recognise the treatable metabolic disturbances. A case of acute intermittent porphyria in a black South African man due to the *HMBS* R149* variant, was reported to mimic severe subacute motor neuropathy [82].

## Conclusion

Although the high rate of consanguinity and occurrence of large families from North Africa have resulted in several molecularly confirmed cases of HSP and CMT, the genetic studies related to identifying the pathogenic variants in these conditions in sub-Saharan Africans, are sparse (Fig. 2). The high proportion of *SMN1*-negative SMA cases in particularly sub-Saharan Africa, identifies another group of patients with an as yet molecularly undiagnosed condition. Although the low rates of genetic reports in these complex disorders are likely due to the lack of resources and limited access to genetic screening, the clinical and genetic characteristics of these disorders need to be described and identified so that the burden of genetic variants and disorders are curated as the first steps to address accessibility to potential therapeutic trials. Collaborations among African researchers are slowly gaining momentum and will strengthen future funding applications to extend specialist clinical training of clinicians and genetic councellors, as well as increasing the number of cases and genomics capabilities in Africa. Increasing neurogenomics capacity and the development of appropriate genetic screening panels for Africans with inherited neuromuscular diseases, would help improve diagnostic capabilities.

## Supplementary Information

**Additional file 1**. **Table A**: Frequencies of genetic variants in African populations with HSP reviewed in gnomAD database. **Table B**: Frequencies of genetic variants in African populations with CMT reviewed in gnomAD database

**Additional file 2**. **Table C**: Reports of autosomal recessive *SMN1*-SMA identified in African populations

## Data Availability

Not applicable.
