# Supplementary material for: A review of the genetic spectrum of hereditary spastic paraplegias, inherited neuropathies and spinal muscular atrophies in Africans
Source: Orphanet J Rare Dis. 2022 Mar 24;17:133. doi: 10.1186/s13023-022-02280-2 (PMC8944057; doi:10.1186/s13023-022-02280-2)
Supplement: Supplementary file 2 — Additional file 2. Table C: Reports of autosomal recessive SMN1-SMA identified in African populations [file 13023_2022_2280_MOESM2_ESM.docx]

**Supplementary Table C: Reports of autosomal recessive *SMN1*-SMA identified in African populations**

| **Reference** | **Country** | **Disease** | **Sample size** | **AAO (years)** | **Homozygous *SMN1* exon 7 ±8 deletion (% of clinical sample)** |
| --- | --- | --- | --- | --- | --- |
| **North Africa** | | | | | |
| (Sifi et al., 2013) | Algeria^+/-^ | SMA I-IV | 92 |  | Exon 7 ± 8 del (75%) |
| (Sbiti et al., 2011) | Morocco^+/-^ | CH  SMA | 60  27 | < 2 | Exon 7 del (38%)  Exon 7 del (78%) |
| (They-They et al., 2008) | Morocco^-^ | SMA I-II | 3 |  | Exon 7 ± 8 del (100%) |
| (Essawi et al., 2007) | Egypt^-^ | SMA I-III | 20 |  | Exon 7 ± 8 del (80%) |
| (Mrad et al., 2006) | Tunisia^-^ | SMA I-IV | 60 | ≤ 2  >2 | Exon 7 del (95%)  Exon 7 ± 8 del (88%) |
| (Bouhouche et al., 2003) | Morocco^+/-^ | SMA I-IV | 54 | ≤ 2  >2 | Exon 7 del (83%)  Exon 7 ± 8 del (63%) |
| (Shawky et al., 2001) | Egypt^-^ | SMA I  SMA II, IV | 21 | ≤ 0.5  >0.5 | Exon 7 del (55%)  Exon 7 ± 8 del (36%) |
| **Sub-Saharan Africa** | | | | | |
| (Vorster et al., 2020) | South Africa: black^-^ | SMA | 197 | NR | Exon 7 ± 8 del (57%) |
| (Labrum et al., 2007) | South Africa: black^-^ | SMA I-III | 115 |  | Exon 7 ± 8 del (35%) |
| Lumaka et al 2009 | Congo^-^ | SMA I | 1 |  | Exon 7 del |
| (Wilmshurst et al., 2002) | South African: black^-^ | SMA I-III | 30 |  | Exon 7 ± 8 del (100%) |
| (Stevens et al., 1999) | South African: black^-^ | SMA | 19 | NR | Exon 7 ± 8 del (65%) |

AAO, Age of Onset; CH, Congenital hypotonia-cause unknown; Hom, homozygous; Het, heterozygous; del, deletion; CNVs, copy number variants; NR, Not reported; +,consanguinity; -, no consanguinity. Blank columns for AAO reflects the SMA I to IV age ranges. Although SMN1- SMA is due to the homozygous SMN1 deletion of exon 7 (± exon 8) we reflect the % of the clinical sample with the genetic confirmation.

References

Bouhouche A, Benomar A, Birouk N, Bouslam N, Ouazzani R, Yahyaoui M, et al. High incidence of SMN1 gene deletion in Moroccan adult-onset spinal muscular atrophy patients. J Neurol 2003;250:1209–13. doi:10.1007/s00415-003-0186-1.

Essawi M, Lk E, Gml S, Gmm A-E, Aa E-H, Am K. Molecular Analysis of SMN1 and NAIP Genes in Egyptian Patients with Spinal Muscular Atrophy. vol. 108. 2007.

Labrum R, Rodda J, Krause A. The molecular basis of Spinal Muscular Atrophy (SMA) in South African black patients. Neuromuscul Disord 2007;17:684–92. doi:10.1016/j.nmd.2007.05.005.

Mrad R, Dorboz I, Ben Jemaa L, Maazoul F, Trabelsi M, Chaabouni M, et al. Molecular analysis of the SMN1 and NAIP genes in 60 Tunisian spinal muscular atrophy patients. Tunis Med 2006;84:465–9.

Sbiti A, Ratbi I, Kriouile Y, Sefiani A. L’amyotrophie spinale infantile: Cause fréquente des hypotonies congénitales au Maroc. Arch Pediatr 2011;18:1261–4. doi:10.1016/j.arcped.2011.09.025.

Shawky RM, Abd El Aleem K, Rifaat MM, Moustafa A. Molecular diagnosis of spinal muscular atrophy in Egyptians. East Mediterr Heal J 2001;7:229–37.

Sifi Y, Sifi K, Boulefkhad A, Abadi N, Bouderda Z, Cheriet R, et al. Clinical and Genetic Study of Algerian Patients with Spinal Muscular Atrophy. J Neurodegener Dis 2013;2013:1–7. doi:10.1155/2013/903875.

Stevens G, Yawitch T, Rodda J, Verhaart S, Krause A. Different molecular basis for spinal muscular atrophy in South African black patients. Am J Med Genet 1999;86:420–6. doi:10.1002/(SICI)1096-8628(19991029)86:5<420::AID-AJMG5>3.0.CO;2-S.

They-They TP, Nadifi S, Dehbi H, Bellayou H, Brik H, Slassi I, et al. Étude de la corrélation génotype-phénotype dans l’amyotrophie spinale infantile (ASI) dans une famille marocaine. Arch Pediatr 2008;15:1201–5. doi:10.1016/j.arcped.2008.04.015.

Vorster E, Essop FB, Rodda JL, Krause A. Spinal Muscular Atrophy in the Black South African Population: A Matter of Rearrangement? Front Genet 2020;11:1. doi:10.3389/fgene.2020.00054.

Wilmshurst JM, Reynolds L, Van Toorn R, Leisegang F, Henderson HE. Spinal muscular atrophy in black South Africans: concordance with the universal SMN1 genotype. Clin Genet 2002;62:165–8. doi:10.1034/j.1399-0004.2002.620210.x.
